# MicroRNAs as regulators of NLRP3 inflammasome activation in herpes simplex virus type 2 infection

**DOI:** 10.3389/fcimb.2025.1602965

**Published:** 2025-05-29

**Authors:** Debashree Dass, Anwesha Banerjee, Ashwini More, Anupam Mukherjee

**Affiliations:** ^1^ ICMR-National Institute of Translational Virology and AIDS Research, Pune, India; ^2^ Savitribai Phule Pune University, Pune, India; ^3^ AcSIR - Academy of Scientific & Innovative Research, Ghaziabad, India; ^4^ ICMR-National Institute of Virology, Pune, India

**Keywords:** HSV-2, inflammation, NLRP3 inflammasome, pyroptosis, caspase-1, IL-1β, mirna regulation

## Abstract

**Introduction:**

Herpes Simplex Virus Type 2 is a prevalent sexually transmitted pathogen that causes genital herpes and severe neurological complications, including meningitis and encephalitis. A major challenge in HSV-2 infection is the uncontrolled inflammatory response mediated by NLRP3 inflammasome activation, leading to pyroptosis and excessive cytokine secretion. Despite its significant clinical burden, the molecular mechanisms underlying HSV-2-induced inflammation remain poorly understood. Recent evidence suggests that microRNAs play a crucial role in regulating host immune responses and inflammasome activation. In this study, we investigate the regulatory role of miR-141 and miR-211 in modulating inflammasome activation and viral replication during HSV-2 infection.

**Methods:**

THP-1-derived macrophages were transfected with miR-141 or miR-211 mimics or scrambled controls before infection with HSV-2. Quantitative PCR and Western blot analysis were performed to assess the expression of NLRP3, CASP1, IL-1β, IL-18, and GSDM-D. Luciferase reporter assays were conducted to validate miRNA–target interactions, and ELISA was used to quantify cytokine levels in culture supernatants.

**Results:**

Our results demonstrate that HSV-2 infection significantly downregulates miR-141 and miR-211, leading to enhanced NLRP3 inflammasome activation, increased caspase-1 cleavage, and excessive secretion of IL-1β and IL-18, ultimately causing pyroptotic cell death. Transfection with miR-141 and miR-211 mimics restored miRNA expression, resulting in a marked suppression of inflammasome activation and inflammatory cytokine release, as well as significant inhibition of HSV-2 viral gene expression. Luciferase assays confirmed that miR-141 directly targets NLRP3, while miR-211 regulates CASP1, validating their roles as post-transcriptional repressors of inflammasome components.

**Discussion:**

These findings establish miR-141 and miR-211 as critical modulators of HSV-2-induced inflammasome activation, highlighting a novel miRNA-based regulatory mechanism. Restoring these miRNAs significantly reduces viral replication and inflammation, underscoring their potential as therapeutic targets for managing HSV-2-induced immunopathology. Future research should focus on *in vivo* validation and therapeutic optimization to develop miRNA-based interventions.

## 1 Introduction

Herpes Simplex Virus Type-2 (HSV-2) is a widespread sexually transmitted infection that primarily causes genital herpes (Jaishankar and Shukla, 2016). Symptoms range from cold sores, blisters, and genital ulcers to severe complications such as vision impairment and neurological disorders (Dass et al., 2023). In particular, HSV-2 can lead to inflammation of the meninges, the protective membranes surrounding the central nervous system and brain, resulting in meningitis (Kupila et al., 2006; Omland et al., 2008). This sexually-transmitted virus impacts approximately 13% of the global adult population, with a higher prevalence among women (Madhivanan et al., 2007; Bauer et al., 2010). It accounts for around 10% of the herpes simplex encephalitis (HSE) cases (Tyler, 2004; Steiner and Benninger, 2013; Bradshaw and Venkatesan, 2016; Ak et al., 2025). Unlike HSV-1, HSV-2-induced encephalitis preferentially affects the brainstem rather than the mesial temporal or orbitofrontal lobes, resulting in a more extensive pattern of inflammation and necrosis (Davis and Johnson, 1979). Despite its significant clinical burden, the molecular mechanisms underlying HSV-2-induced inflammation and immune evasion strategies remain incompletely understood.

Host immune responses to HSV-2 involve the activation of innate immune signaling pathways, particularly through pattern recognition receptors (PRRs) that detect viral proteins and initiate inflammatory cascades (Zheng et al., 2023). A key component of this response is the NOD-like receptor family pyrin domain-containing 3 (NLRP3) inflammasome, a multiprotein complex crucial for innate immune defense (Xu and Núñez, 2023). Upon activation, NLRP3 recruits the adaptor protein ASC, leading to the cleavage of pro-caspase-1 into its active form (CASP1), which subsequently processes the precursor forms of pro-inflammatory cytokines interleukin-1β (IL-1β) and interleukin-18 (IL-18) into their biologically active states. These cytokines are then secreted, amplifying inflammatory responses and contributing to host defense (Dinarello, 2011; Strowig et al., 2012). While this inflammatory response plays a protective role in early infection, excessive or prolonged cytokine release can cause collateral tissue damage, exacerbating the disease (Menu and Vince, 2011; Chen et al., 2018; Marcocci et al., 2020). In some cases, the inflammatory response culminates in pyroptosis, a highly inflammatory form of programmed cell death driven by Gasdermin-D (GSDM-D) activation, leading to membrane rupture and further amplification of the immune response (Walsh et al., 2014; Man, 2018). Although HSV-1 has been extensively studied in this context, the precise mechanisms through which HSV-2 activates and modulates inflammasome signaling remain unclear.

Recent evidence suggests that microRNAs (miRNAs), small non-coding RNA molecules that regulate gene expression, play a crucial role in the modulating host immune responses (Moens, 2009; Piedade and Azevedo-Pereira, 2016; Hussein and Akula, 2017; Dass et al., 2023). Dysregulation of specific miRNAs has been implicated in various infectious diseases, including herpesvirus infections (Hum et al., 2021; Banerjee et al., 2023). Studies have shown that viral infections can manipulate host miRNA expression to evade immune detection and establish persistent infection (Diener et al., 2022). In the case of HSV-2, emerging data indicate that certain miRNAs may regulate inflammasome activity, thus influencing the outcome of infection. MiRNA-based therapeutics offer a promising avenue for modulating inflammatory responses with potentially fewer side effects compared to conventional antiviral therapies, as miRNAs are endogenous cellular components with specific gene-targeting capabilities (Diener et al., 2022).

In this study, we investigate the molecular interplay between HSV-2 infection and inflammasome activation, with a specific focus on miRNA-mediated regulation. We identify miR-141 and miR-211 as key regulators of inflammasome signaling and demonstrate that their downregulation upon HSV-2 infection intensifies NLRP3 inflammasome activation, leading to increased inflammatory cytokine secretion and pyroptotic cell death. By ectopically expressing the miRNAs, miR-141 and miR-211, we show a significant suppression of inflammasome activation and HSV-2 replication. These findings provide critical insights into HSV-2-induced inflammation and highlight the potential of miRNA-based therapeutics as a novel strategy to mitigate HSV-2-associated complications.

## 2 Materials and methods

### 2.1 Cell culture

The inflammatory experiments and infection assays were conducted on the human monocyte/macrophage-like cell line THP-1 (ATCC: TIB-202™, ATCC, Manassas, VA, USA) while the HEK 293T (Human Embryonic Kidney) cells (ATCC: CRL-3216™, ATCC, Manassas, VA, USA) were used for *in vitro* validation. Vero epithelial cells (ATCC: CCL-81™) were utilized for HSV-2 propagation (ATCC: VR-734D™, ATCC, Manassas, VA, USA). HEK 293T and Vero cells were maintained in Dulbecco’s Modified Eagle Medium (DMEM) (Gibco, Waltham, MA, USA), while THP-1 cells were cultured in RPMI-1640 medium (Gibco, Waltham, MA, USA). All media were supplemented with 10% fetal bovine serum (FBS) (Invitrogen, Waltham, MA, USA), 20 mM HEPES, and 1% penicillin-streptomycin. Additionally, THP-1 cells were supplemented with 1 mM sodium pyruvate. THP-1 cells were differentiated into macrophages using 10 nM Phorbol 12-myristate 13-acetate (PMA; Sigma-Aldrich, St. Louis, MO, USA) for 24 hours, followed by a 24-hour rest period in PMA-free media. All cell cultures were maintained at 37°C in a 5% CO_2_ humidified atmosphere, regularly monitored for mycoplasma contamination, and handled according to ATCC guidelines.

### 2.2 Virus propagation

HSV-2 (ATCC: VR-734D™, ATCC, Manassas, VA, USA) was propagated in Vero cells under serum-starved conditions with 2% FBS for 2–3 days. Viral titers were quantified using a standard plaque assay technique (Huleihel et al., 2001).

### 2.3 SYBR-green quantitative PCR

HSV-2 infection was given at MOI 1 while LPS treatment was administered at the concentration of 10μg/ml for 24 hours. Total RNA was extracted using the TRIzol (Invitrogen, Waltham, MA, USA) organic extraction method, and cDNA was synthesized via reverse transcription using the SuperScript™ III First-Strand Synthesis System (Invitrogen: 18080-051). Infection was confirmed by amplifying the HSV-2 viral gene UL30 using Power SYBR™ Green PCR Master Mix (Applied Biosystems™, Madison, WI, USA: 4367659) with 200 nM UL30-specific primers. The reduction in viral mRNA levels (UL30, RL2, and UL44) was assessed post-transfection with miRNA mimics. All qPCR experiments adhered to MIQE guidelines, with GAPDH as the internal reference for normalization. Relative changes in mRNA levels were calculated using the 2^−ΔΔCt^ method, and results were expressed as fold changes relative to controls. Primer sequences are listed in **Supplementary Table S1**.

### 2.4 Enzyme-linked immunosorbent assay

Cell supernatants were collected at designated time points to measure IL-1β levels using the Human IL-1β ELISA Kit (BMS224-2, Invitrogen, Waltham, MA, USA) and IL-18 levels using the Human IL-18 ELISA Kit (DBP180, R&D Systems, Minneapolis, MN, USA) as per the manufacturers’ instructions. Briefly, samples were applied to ELISA plates pre-coated with IL-1β- or IL-18- specific antibodies, followed by detection antibodies and substrates. Absorbance was measured at the specified wavelength to quantify cytokine levels.

### 2.5 miRNA target prediction

The miRNAs hsa-miR-141-3p and hsa-miR-211-5p were selected for this study based on preliminary screening using the Human Inflammatory and Immune Response miRNA PCR Array (Qiagen, Cat. No. MIHS-105ZA), which revealed their downregulation in HSV-2-infected THP-1 cells (Banerjee et al., 2023). These miRNAs are the mature, functional strands derived from the 3′ arm of pre-miR-141 and the 5′ arm of pre-miR-211, respectively, as annotated in miRBase (release 22.1). To identify putative targets relevant to inflammasome activation, we performed *in silico* predictions using three independent databases: miRWalk (http://mirwalk.umm.uni-heidelberg.de/ accessed on July 12, 2022), TargetScanHuman 8.0 (https://www.targetscan.org/vert_80/ accessed on January 25, 2023), miRDB (http://mirdb.org/ accessed on January 29, 2023) (Agarwal et al., 2015; Sticht et al., 2018; Chen and Wang, 2020). These analyses consistently predicted NLRP3 as a target of hsa-miR-141-3p and CASP1 as a target of hsa-miR-211-5p. The seed region complementarity and binding interactions for these targets were visualized based on TargetScanHuman 8.0 output. The combination of predictions from multiple platforms strengthened the confidence in selecting these target genes for experimental validation.

### 2.6 TaqMan-based quantitative PCR for miRNA validation

To validate miRNAs involved in the inflammatory pathway, TaqMan-based quantitative PCR was employed. miRNAs from HSV-2-infected THP-1 cells were extracted using the mirVana™ miRNA Isolation Kit (Thermo Fisher Scientific, Cat. No. AM1560). Specific miRNAs were validated using TaqMan primer-probe assays for hsa-miR-141-3p (Assay ID: 000463) and hsa-miR-211-5p (Assay ID: 00051674). Reverse transcription and real-time PCR were performed using the TaqMan™ MicroRNA Reverse Transcription Kit, optimized for low RNA input. RNA-U6 was used as the endogenous control for normalization. Relative miRNA expression was calculated using the 2^−ΔΔCt^ method, with graphical representation highlighting miRNA expression dynamics.

### 2.7 Cloning and transfection

The 3′ UTR regions of NLRP3 and CASP1 genes, involved in inflammasome assembly and predicted as targets of miR-141 and miR-211, were amplified using gene-specific primers (**Supplementary Table S2**) with MluI and HindIII restriction sites. PCR amplicons and pMIR-REPORT luciferase vector (Ambion, Austin, TX, USA: AM5795) were digested with MluI and HindIII (New England Biolabs, Rowley, MA, USA: R0198S and R0104S) and ligated using T4 DNA Ligase (New England Biolabs, M0202S). Ligation products were transformed into DH5α competent cells, and recombinant plasmids were extracted using the QIAprep Spin Miniprep Kit (Qiagen, Hilden, Germany: 27106).

The recombinant plasmids containing the cloned 3′ UTR regions were then transfected or co-transfected into HEK293T or THP-1 cells using Lipofectamine 2000 (Invitrogen, Waltham, MA, USA: 11668019) following the manufacturer’s instruction. Functional studies utilized miRNA mimics (50 nM) for miR-141-3p (MC10860) and miR-211-5p (MC10168), along with scrambled miR control obtained from Thermo Fisher Scientific (Waltham, MA, USA).

### 2.8 Luciferase reporter assay

To validate miRNA targets, wild-type and mutated 3′ UTRs of NLRP3 and CASP1 were cloned into pMIR-REPORT plasmids and transfected into HEK293T cells along with miRNA mimics or scrambled miR controls. Luciferase activity was measured using the Luciferase Reporter Gene Detection Kit (Sigma-Aldrich, LUC1-1KIT). Reduced luciferase activity confirmed miRNA binding to target genes.

### 2.9 Western blot

Cells were harvested, and lysates were prepared using a lysis buffer supplemented with protease inhibitors to maintain protein integrity. The lysates were subjected to protein separation via electrophoresis on a 10% polyacrylamide gel, followed by transfer onto a PVDF membrane. The membrane was blocked with 5% non-fat dried milk for 1 hour at room temperature and incubated with primary antibodies specific to NLRP3 (15101), ASC (13833), Cleaved CASP1 (p20 subunit - Asp297; 4199), IL-1β (12703), IL-18 (57058), Cleaved GSDMD (Asp275; 36425) procured from Cell Signaling Technology (Danvers, MA, USA), HSV-2 gB (57857; Santa Cruz Biotechnology, Santa Cruz, CA, USA) and HSV-2 ICP8 (56992; Santa Cruz Biotechnology, Santa Cruz, CA, USA). The proteins bound to the primary antibodies were detected using either horseradish peroxidase (HRP)-conjugated primary or secondary antibodies. Signals were visualized using Pierce™ ECL Western Blotting Substrate (Thermo Scientific, Waltham, MA, USA), and chemiluminescence was captured using a ChemiDoc imaging system (Bio-Rad, Hercules, CA, USA). The membrane was stripped using 1× ReBlot Plus Strong Antibody Stripping Solution (Merck-Millipore, Burlington, MA, USA: 2504), re-blocked, and re-probed with a GAPDH antibody (Santa Cruz Biotechnology, Santa Cruz, CA, USA: 47724-HRP) as an internal loading control. Protein band intensities were quantified using ImageJ software (NIH, version 1.53a) and normalized to GAPDH levels for comparative analysis.

### 2.10 Statistical analysis

Statistical significance was determined using GraphPad Prism v9.0. Data were analyzed using one-way ANOVA or Student’s t-test, and results were presented as mean ± standard deviation (SD) from at least three independent experiments. *P*-values < 0.05 were considered statistically significant.

## 3 Results

### 3.1 HSV-2 activates inflammation through the NLRP3 pathway

Upon viral infection, pattern recognition receptors (PRRs) sense viral entry and trigger inflammatory responses (Li and Wu, 2021). While HSV-1 is known to upregulate NLRP3 inflammasome activation, the mechanism by which HSV-2 mediates inflammation remains unclear (Li et al., 2019; Vitali et al., 2020). Given that NLRP3 is the most studied inflammasome in viral infections (Zheng et al., 2023), we investigated whether HSV-2 triggers inflammation through NLRP3 inflammasome activation in THP-1-derived macrophages.

**Figure 1:**
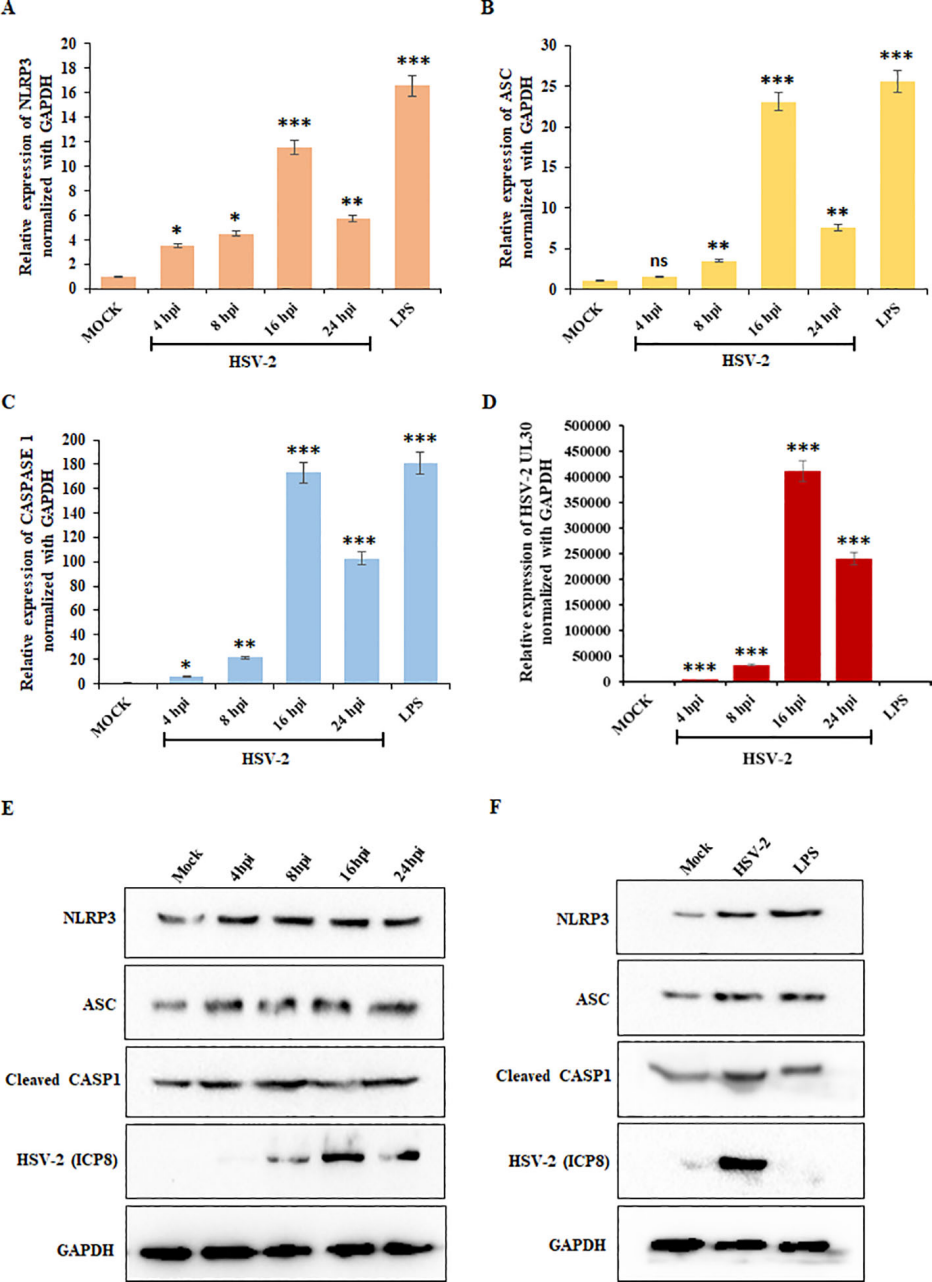
HSV-2 infection triggers inflammatory responses: Graphical representation of the results of the qRT-PCR analysis showing relative mRNA expression levels of **(A)** NLRP3, **(B)** ASC, **(C)** CASP1, and **(D)** UL30 in THP-1 cells infected with HSV-2 (MOI = 1) at 4-, 8-, 16-, and 24-hpi, after normalization with the internal control, GAPDH. Mock-infected cells served as a negative control, while lipopolysaccharides (LPS) treatment was used as a positive control. **(E)** Immunoblot analysis demonstrating the post-infection kinetics of the protein expression levels of NLRP3, ASC, Cleaved CASP1, HSV-2 ICP8, and GAPDH in THP-1 cells at 4-, 8-, 16-, and 24-hpi compared to the mock-infected samples. **(F)** Comparison of the protein expression levels of NLRP3, ASC, Cleaved CASP1, HSV-2 ICP8, and GAPDH in mock-infected, HSV-2 infected (MOI=1) and uninfected, LPS-treated THP-1 cells at 24 hours. GAPDH was used as a loading control for normalization. Data represent the mean ± SD of three independent experiments. Statistical analysis was performed using one-way ANOVA followed by Tukey’s multiple comparisons test. The number of asterisks, as shown in each of the graphs, indicates the level of significance of the data (*p<0.05, **p<0.01, ***p<0.001).

To determine the expression of key inflammasome components, we performed quantitative PCR for NLRP3, ASC, and CASP1 – the essential inflammasome components, along with UL30 to confirm HSV-2 infection. THP-1 cells were infected with HSV-2 at 1 MOI, and samples were collected at 4-, 8-, 16-, and 24-hours post-infection (hpi). LPS-treated cells served as a positive control. A significant increase in NLRP3 mRNA expression was observed with progressing infection, peaking at 16 hpi (11-fold increase compared to mock-infected cells) (**Figure 1A**). ASC mRNA expression also increased significantly at 16 hpi (23-fold increase), followed by declining at 24 hpi while remaining elevated compared to earlier time points (**Figure 1B**). CASP1 mRNA levels showed a dramatic upregulation, peaking at 16 hpi (170-fold increase) and sustaining at 24 hpi (**Figure 1C**). Meanwhile, UL30 mRNA levels increased significantly at 16 hpi (**Figure 1D**). The inflammatory receptor NLRP3 and its downstream components, ASC and CASP1, exhibited peak mRNA expression at 16 hpi, which then declined by 24 hpi (**Figures 1A-C**), suggesting temporally regulated transcriptional and translational dynamics.

To validate these findings at the protein level, we performed Western blot analysis for NLRP3, ASC, Cleaved CASP1, HSV-2 ICP8, and GAPDH at different time points as indicated (**Figure 1E**). Compared to mock-infected cells, HSV-2 infection led to an increase in NLRP3, ASC, and cleaved CASP1 protein levels, with quantification confirming peak expression at 24 hpi (**Supplementary Figure S1**), confirming NLRP3 inflammasome activation upon HSV-2 infection. LPS-treated cells exhibited robust activation of inflammasome markers, confirming the validity of the experimental setup. HSV-2 infection at 24 hpi led to similar protein expression of the inflammatory markers as the 24 hours-LPS-treated cells (**Figure 1F**; **Supplementary Figure S2**). Additionally, the HSV-2 ICP8 protein, a marker of viral replication, showed a consistent increase over time, peaking at 24 hpi, confirming viral progression. These findings confirm that mRNA expression of inflammatory markers peaks at 16 hpi, followed by significant protein expression at 24 hpi, reflecting a transition from transcriptional activation to translational regulation. Overall, these results suggest that HSV-2 infection triggers NLRP3 inflammasome activation, leading to increased inflammatory responses.

### 3.2 HSV-2-induced inflammation triggers pyroptosis

The activation of inflammasome plays an important role in the innate immune response to infections, including their involvement in pyroptosis, a pro-inflammatory form of programmed cell death (Guo et al., 2015). Inflammasome activation often leads to the cleavage of Gasdermin-D (GSDM-D) and subsequent secretion of inflammatory cytokines, such as IL-1β and IL-18, which amplify inflammatory responses (Yao et al., 2024). HSV-1 has been shown to induce pyroptosis, thereby exacerbating inflammation (Hu et al., 2022; Jiang et al., 2024). Similarly, HSV-2 has also been shown to induce cell death, which may further contribute to inflammation (Vitali et al., 2020; Dass et al., 2024). Yet the mechanisms driving HSV-2-induced pyroptosis remain unexplored. To investigate whether HSV-2 infection induces pyroptosis, we analyzed the expression of IL-1β, IL-18, and GSDM-D at the mRNA and protein levels in HSV-2-infected THP-1 cells.

**Figure 2:**
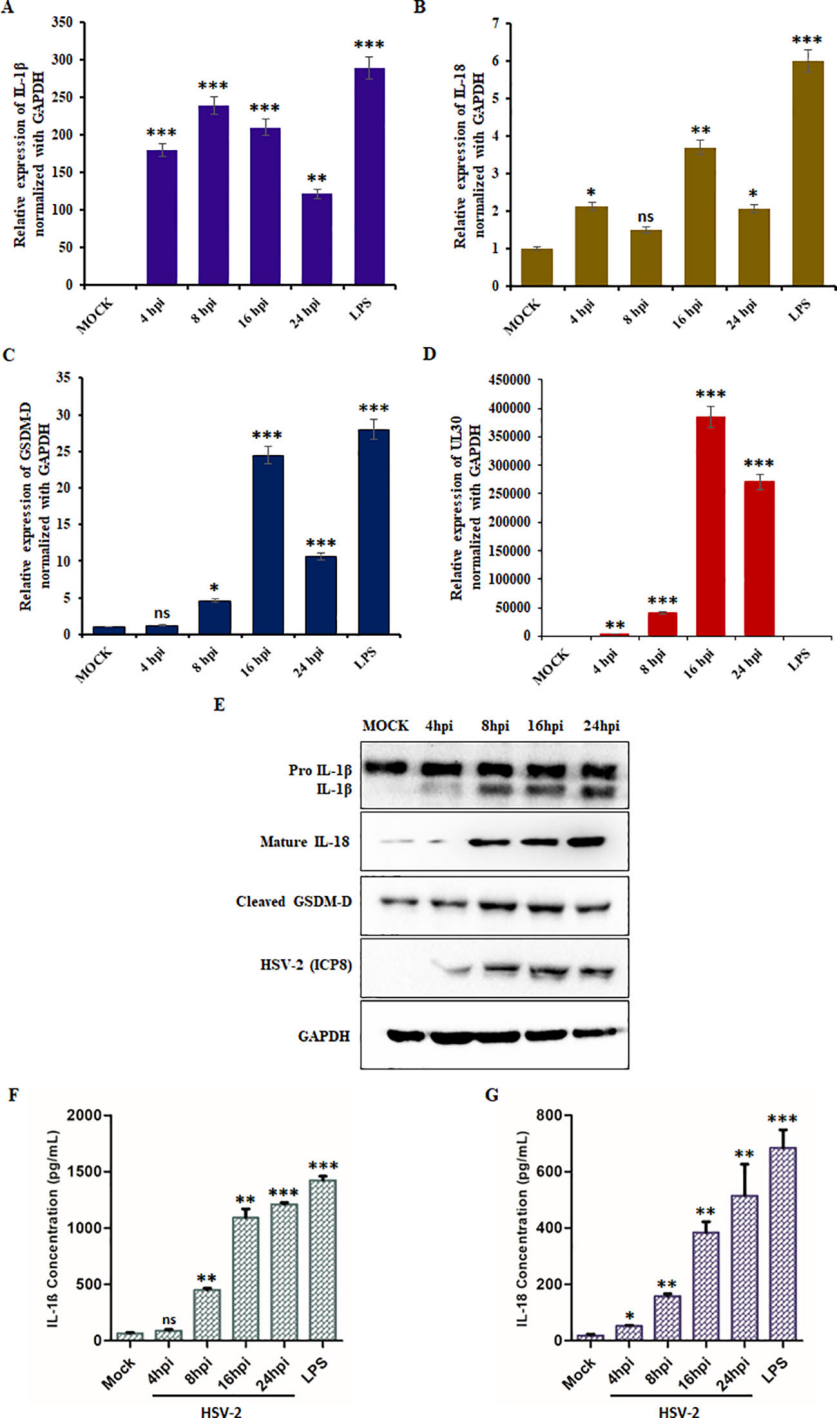
HSV-2 trigger inflammation-induced pyroptosis: Graphical representation of relative fold change in mRNA expression levels of **(A)** IL-1β, **(B)** IL-18, **(C)** GSDM-D, and **(D)** HSV-2 UL30 in THP-1 cells infected with HSV-2 (MOI = 1) at the indicated time points (4-, 8-, 16-, and 24- hpi). Mock-infected cells served as a negative control, and LPS-treated cells were used as a positive control. **(E)** Immunoblot analysis showing the protein levels of pro-IL-1β, active IL-1β, active IL-18, cleaved GSDM-D, HSV-2 ICP8, and GAPDH in mock-infected and HSV-2 (MOI= 1) infected THP-1 cells at 4-, 8-, 16-, and 24-hpi. GAPDH was used as a loading control for normalization. Quantification of secreted **(F)** IL-1β and **(G)** IL-18 levels in the culture supernatants of THP-1 cells that were mock-infected/infected with HSV-2 at the indicated time points/LPS-treated, as measured by ELISA. Data represent the mean ± SD of three independent experiments. Statistical analysis was performed using one-way ANOVA followed by Tukey’s multiple comparisons test. The number of asterisks, as shown in each of the graphs, indicates the level of significance of the data (*p<0.05, **p<0.01, ***p<0.001).

qPCR analysis revealed a significant increase in IL-1β, IL-18, and GSDM-D mRNA expression upon HSV-2 infection (**Figures 2A-C**). IL-1β levels increased steadily, reaching a 200-fold change at 16 hpi (**Figure 2A**), while IL-18 expression peaked at 16 hpi (4-fold increase) before declining at 24 hpi (**Figure 2B**). Similarly, GSDM-D mRNA levels exhibited peak upregulation at 16 hpi (25-fold increase), suggesting activation of the pyroptotic pathway (**Figure 2C**). UL30 expression continued to increase, confirming active HSV-2 replication (**Figure 2D**).

To confirm these results, we performed Western blot analysis for IL-1β, IL-18, and GSDM-D in infected THP-1 cells (**Figure 2E**). Protein expression of mature IL-1β and IL-18 progressively increased over time (**Supplementary Figure S3**). Additionally, the cleavage of GSDM-D, a hallmark of pyroptotic cell death, was detected, with levels increasing upon HSV-2 infection (**Supplementary Figure S3**). Furthermore, analysis of culture supernatants from HSV-2-infected THP-1 cells revealed a marked increase in IL-1β and IL-18 secretion, peaking at approximately 1200 pg/mL and 550 pg/mL, respectively, at 24 hpi (**Figures 2F-G**).

Collectively, these findings demonstrate that HSV-2-induced inflammasome activation leads to pyroptosis, as indicated by increased IL-1β and IL-18 expression and release, along with the proteolytic cleavage of GSDM-D. These data highlight pyroptosis as a key component of HSV-2 pathogenesis.

### 3.3 miR-141 and miR-211 are downregulated during HSV-2 infection

miRNAs serve as key regulators of inflammation and immune responses, and recent studies suggest their involvement in HSV-2 pathogenesis (Wang et al., 2022; Banerjee et al., 2023; Yan et al., 2023; Long et al., 2024; Zhan et al., 2024). Among various miRNAs associated with immune modulation, miR-141 and miR-211 were selected based on preliminary array screening of HSV-2-infected macrophages, which revealed significant downregulation of these miRNAs (Banerjee et al., 2023). Both are predicted to target core components of the NLRP3 inflammasome pathway (NLRP3 and CASP1, respectively), and prior studies have reported their roles in suppressing inflammatory responses and pyroptosis (Wang et al., 2022; Yan et al., 2023; Long et al., 2024; Zhan et al., 2024). Given the strong association between HSV-2 infection and excessive inflammation, we sought to determine whether HSV-2 modulates miR-141 and miR-211 expression, potentially contributing to viral pathogenesis.

**Figure 3:**
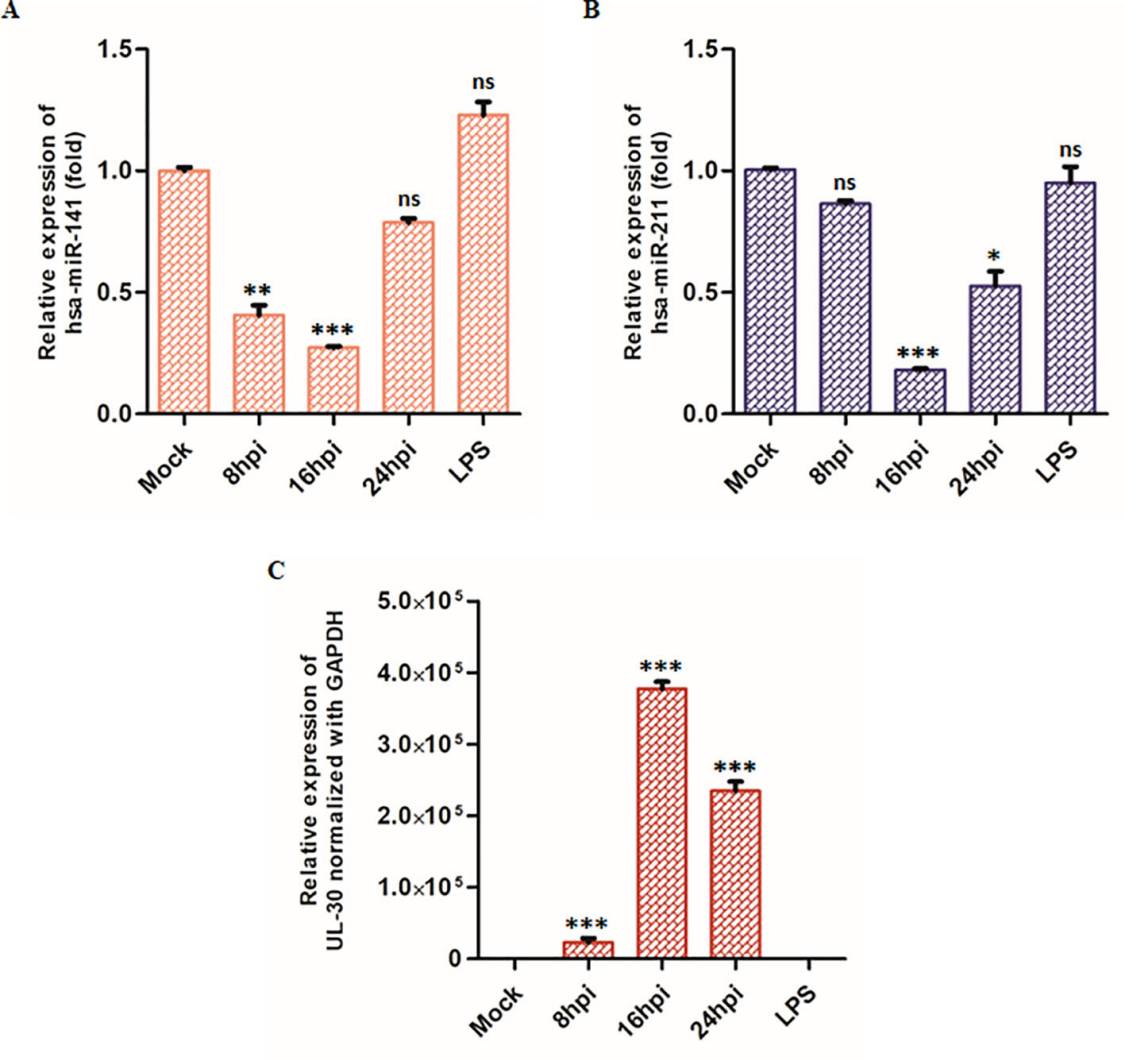
TaqMan Assay of the target miRNAs: Graphical representation of **(A)** miR-141, **(B)** miR-211 miRNA and **(C)** HSV-2 UL30 mRNA expression during HSV-2 infection at 8-, 16- and 24 hpi relative to mock-infected cells. LPS-treatment (10 µg/mL, 24 hours) were included as an additional control to assess the specificity of miRNA downregulation during HSV-2 infection. miR-141 and miR-211 levels were quantified using specific TaqMan assays and normalized to RNA-U6. HSV-2 UL30 expression was measured by SYBR Green qPCR and normalized to GAPDH. Data represent the mean ± SD of at least three independent experiments. Statistical analysis was performed using one-way ANOVA followed by Tukey’s multiple comparisons test. The number of asterisks indicates the level of significance (*p<0.05, **p<0.01, ***p<0.001).

To assess the expression dynamics of these miRNAs during HSV-2 infection, we performed TaqMan-based qPCR on total RNA extracted from HSV-2-infected THP-1 macrophages at 8-, 16-, and 24 hpi. Our qPCR analysis demonstrated a significant downregulation of both miRNAs miR-141 and miR-211 upon HSV-2 infection, with distinct temporal patterns (**Figures 3A, B**). At 8 hpi, miR-141 expression was markedly reduced to 0.40-fold compared to mock-infected controls, indicating a substantial suppression of miR-141 during the early phase of infection (**Figure 3A**). Conversely, miR-211 expression showed no significant change at this time point (**Figure 3B**). At 16 hpi, both miRNAs were significantly downregulated. By 24 hpi, miR-141 expression showed partial recovery (**Figure 3A**), whereas miR-211 levels exhibited a more pronounced decline, reaching 0.48-fold of mock-infected controls (**Figure 3B**), suggesting enhanced suppression during the later stage of infection. Notably, LPS treatment did not significantly alter miR-141 or miR-211 expression levels (**Figures 3A, B**), indicating that their downregulation is specific to HSV-2 infection rather than a general inflammatory response. These findings suggest that miR-141 downregulation occurs predominantly during early HSV-2 infection, whereas miR-211 suppression intensifies at later stages, possibly reflecting differential miRNA regulation as viral replication progresses. UL30 mRNA expression, a viral gene encoding the HSV-2 DNA polymerase catalytic subunit, was used as an indicator of viral replication kinetics across these time points (**Figure 3C**).

### 3.4 miR-141 and miR-211 regulate inflammation by targeting NLRP3 and CASP1

Given the significant downregulation of miR-141 and miR-211 during HSV-2 infection, we sought to determine whether these miRNAs regulate key inflammatory pathways associated with HSV-2-induced immune responses. Using *in silico* analysis, we employed the miRWalk database (http://mirwalk.umm.uni-heidelberg.de/, accessed on July 12, 2022) to predict potential target genes of miR-141 and miR-211 that may be involved in inflammasome activation. Our analysis identified NLRP3 and CASP1 as putative targets of miR-141 and miR-211, respectively. These genes encode key components of the NLRP3 inflammasome, a critical regulator of inflammatory responses leading to IL-1β and IL-18 maturation and pyroptosis.

**Figure 4:**
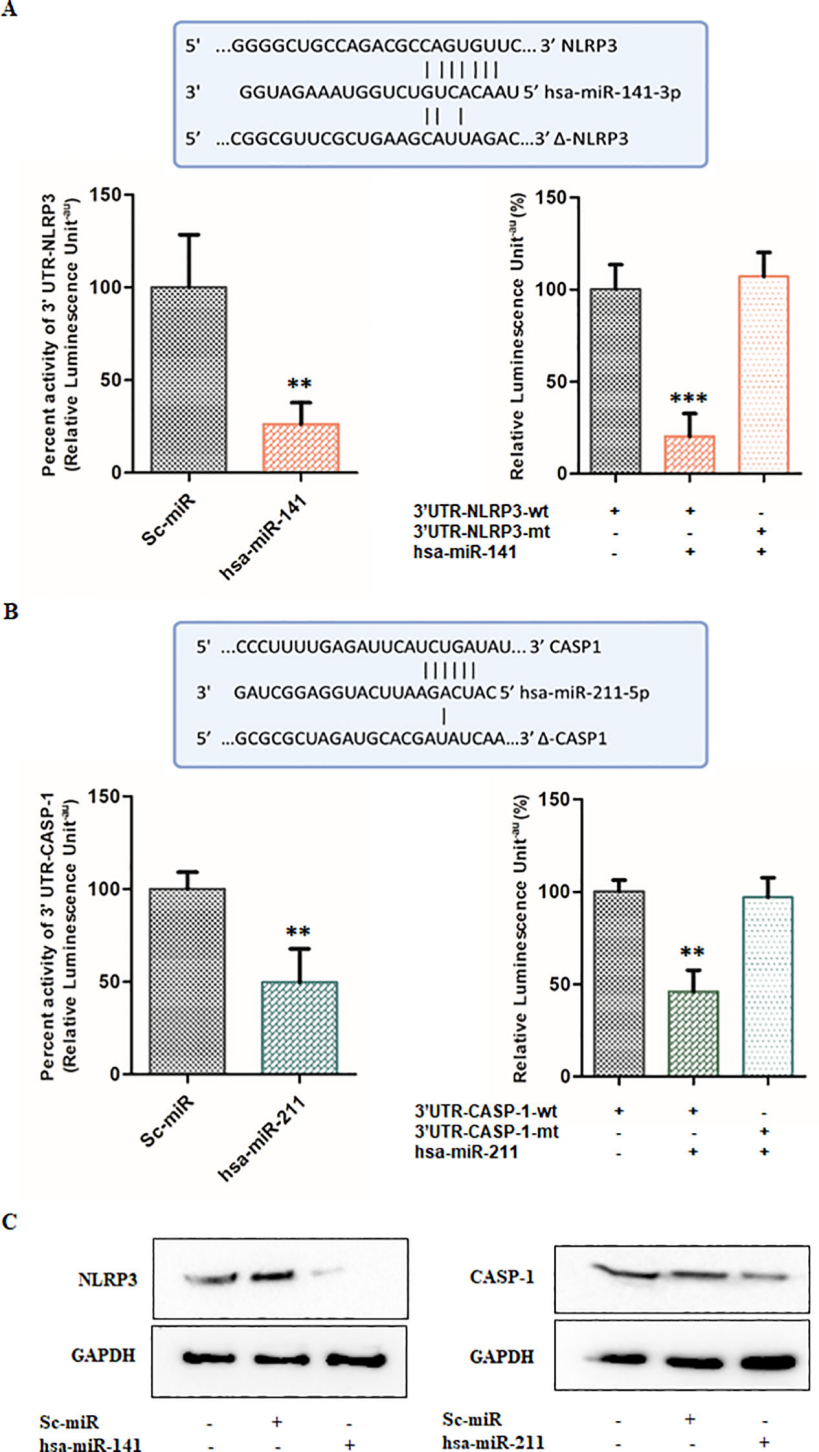
miR-141 and miR-211 regulate inflammation by targeting NLRP3 and CASP1, respectively, during HSV-2 infection: **(A)** Target validation of NLRP3 by miR-141 and **(B)** Target validation of CASP1 by miR-211. The complementary sequences between the seed regions of the wild-type and mutant (Δ) 3′ UTRs of NLRP3 and CASP1 with the respective miR-141 and miR-211 binding sites are shown. Relative luciferase activity (%) was measured in HEK293T cells transfected with miRNA target/co-transfected with miRNA target and scrambled miRNAs or miRNA mimic of interest, to demonstrate target regulation. Also, cells were transfected with wild-type (wt) or mutated (mt) 3′ UTRs of NLRP3 and CASP1 along with respective miRNA mimics to validate sequence-specific targeting by miRNAs. **(C)** Immunoblot analysis of NLRP3 and CASP1 protein levels following transfection with miR-141 and miR-211 mimics or scrambled controls. GAPDH served as the loading control. Data represent the mean ± SD of three independent experiments.The number of asterisks, as shown in each of the graphs, indicates the level of significance of the data (**p<0.01, ***p<0.001).

To validate these computational predictions, we performed a luciferase reporter assay using wild-type and mutated 3′ UTR sequences of NLRP3 and CASP1 cloned into the pMIR-REPORT luciferase vector. HEK293T cells were co-transfected with either miR-141 or miR-211 mimics, along with the corresponding wild-type or mutated 3′ UTRs of NLRP3 or CASP1. Scrambled miRNA controls (sc-miR) were used as negative controls to account for non-specific effects. Our results demonstrated a significant suppression of luciferase activity upon co-transfection with wild-type NLRP3 and miR-141 mimics, indicating a direct interaction between miR-141 and NLRP3 (**Figure 4A**). Specifically, luciferase activity was reduced by 74-80% in co-transfected cells containing the wild-type NLRP3 3′ UTR, whereas no significant reduction was observed in co-transfected cells containing the mutated NLRP3 3′ UTR, confirming the specificity of miR-141–NLRP3 interaction (**Figure 4A**). Similarly, co-transfection of miR-211 mimics with wild-type CASP1 3′ UTR resulted in a 50-54% decrease in luciferase activity compared to control cells, whereas the mutated CASP1 3′ UTR exhibited no significant change in luciferase activity (**Figure 4B**). These findings validate that miR-141 directly targets NLRP3, while miR-211 targets CASP1, establishing their functional roles in regulating inflammasome activation (**Figures 4A, B**). To further confirm the regulatory effects of miR-141 and miR-211, we assessed NLRP3 and CASP1 protein levels in THP-1 macrophages transfected with miRNA mimics or scrambled controls. Western blot analysis revealed that transfection with miR-141 mimic led to a substantial decrease in NLRP3 protein expression (**Figure 4C** – left panel), whereas miR-211 mimic transfection resulted in a notable reduction in CASP1 protein levels (**Figure 4C** – right panel). Scrambled miRNA controls had no effect on NLRP3 or CASP1 expression, further supporting the specificity of miR-141 and miR-211 in regulating inflammasome components.

Taken together, these results establish miR-141 and miR-211 as key post-transcriptional regulators of inflammasome activation, directly targeting NLRP3 and CASP1, respectively. The suppression of these miRNAs upon HSV-2 infection likely contributes to enhanced inflammasome activation, increased IL-1β and IL-18 secretion, and exacerbated inflammation. These findings indicate that miR-141 and miR-211 act as negative regulators of HSV-2-induced inflammatory responses and suggest their potential as therapeutic targets for controlling excessive immune activation during HSV-2 infection.

### 3.5 Ectopic expression of miR-141 and miR-211 suppresses HSV-2 replication

**Figure 5:**
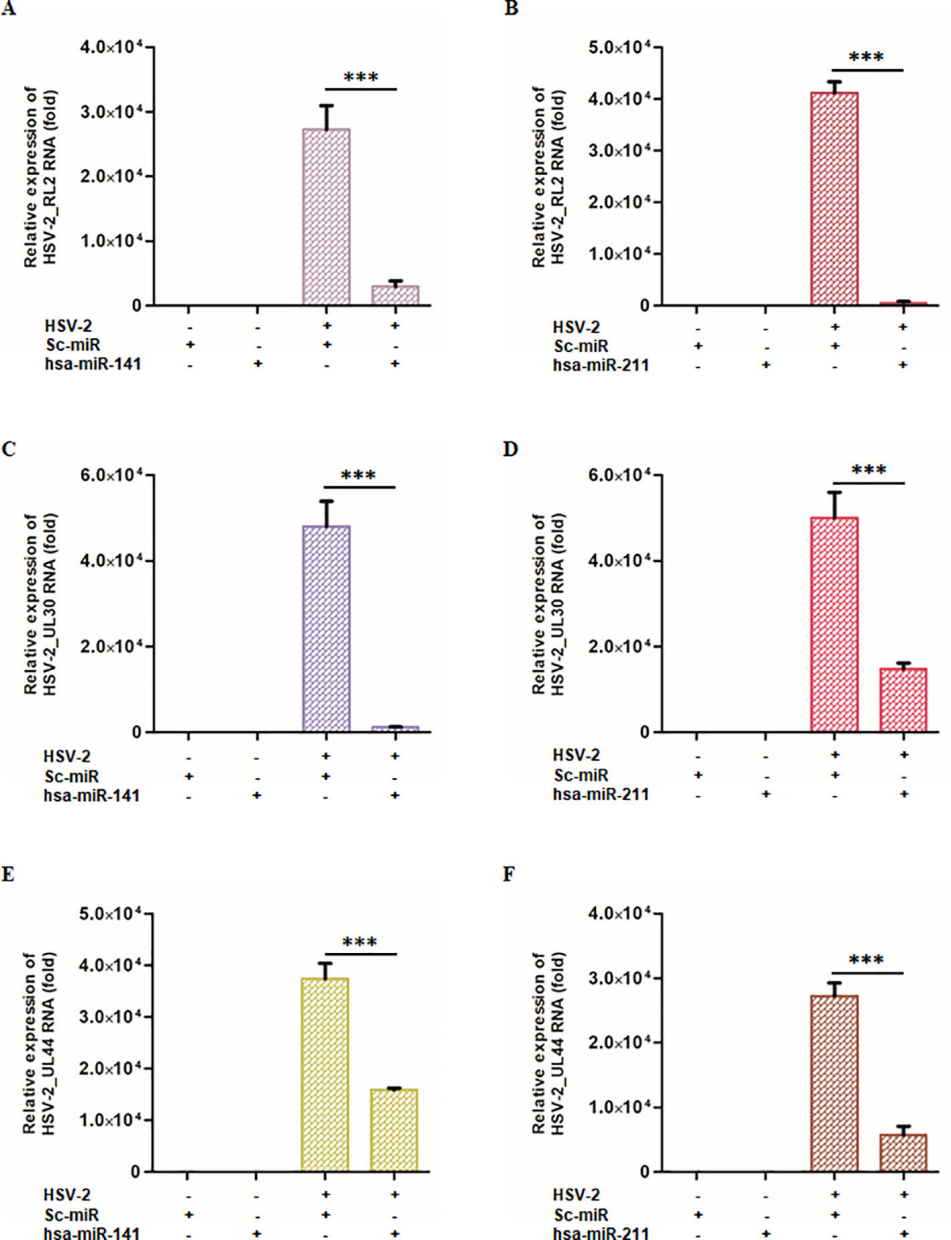
miR-141 and miR-211 modulates HSV-2 viral gene expression across different replication phases: THP-1-derived macrophages were transfected with either miR-141, miR-211, or scrambled control miRNA, followed by HSV-2 infection. At 8 hpi, viral gene expression was analyzed across different replication phases by measuring key viral transcripts: RL2 (immediate early phase), UL30 (early phase), and UL44 (late phase). **(A-C)** The effects of miR-141 on HSV-2 viral gene transcripts. miR-141 significantly reduced the expression of all viral genes in the HSV-2 infected cells. **(D-F)** The effects of miR-211 under identical experimental conditions. Similar to miR-141, miR-211 demonstrated potent antiviral activity. “-” and “+” indicate absence or presence of HSV-2, sc-miR, miR-211, or miR-141 as specified. Data represent the mean ± SD of at least three independent experiments. Statistical analysis between two groups was performed using an unpaired two-tailed Student’s t-test (***p < 0.001).

Having established miR-141 and miR-211 as key regulators of NLRP3 and CASP1, we next investigated whether their ectopic expression could mitigate HSV-2 replication. THP-1-derived macrophages were transfected with either miR-141 or miR-211 mimics (50 nM) or a scrambled miRNA (sc-miR) control prior to infection with HSV-2 (MOI = 1). At 8 hpi, the expression levels of key HSV-2 viral genes (RL2, UL30, and UL44) were quantified by qPCR (**Figure 5**).

Quantitative PCR analysis revealed a significant reduction in viral gene expression upon miR-141 or miR-211 mimic transfection. In miR-141-transfected macrophages, RL2 expression was reduced by 89% compared to sc-miR-transfected cells (**Figure 5A**), while miR-211-transfected macrophages showed a 98.6% reduction (**Figure 5B**). Similarly, UL30 expression was suppressed by 97.4% and 70.5% following miR-141 and miR-211 mimic transfection, respectively (**Figures 5C, D**). UL44 expression was also substantially reduced by 57.5% and 78.8% in miR-141- and miR-211-transfected cells, respectively (**Figures 5E, F**). To assess the impact on infectious virion production, culture supernatants from miRNA mimic-transfected, HSV-2-infected THP-1 cells were analyzed by plaque assay. A 70–85% reduction in infectious virus titers was observed compared to sc-miR controls (**Supplementary Figure S4**). These findings demonstrate that miR-141 and miR-211 interfere with multiple stages of the HSV-2 replication cycle, highlighting their potential as antiviral therapeutic candidates.

### 3.6 Ectopic expression of miR-141 and miR-211 attenuates HSV-2-induced inflammasome activation and pyroptotic inflammation

**Figure 6:**
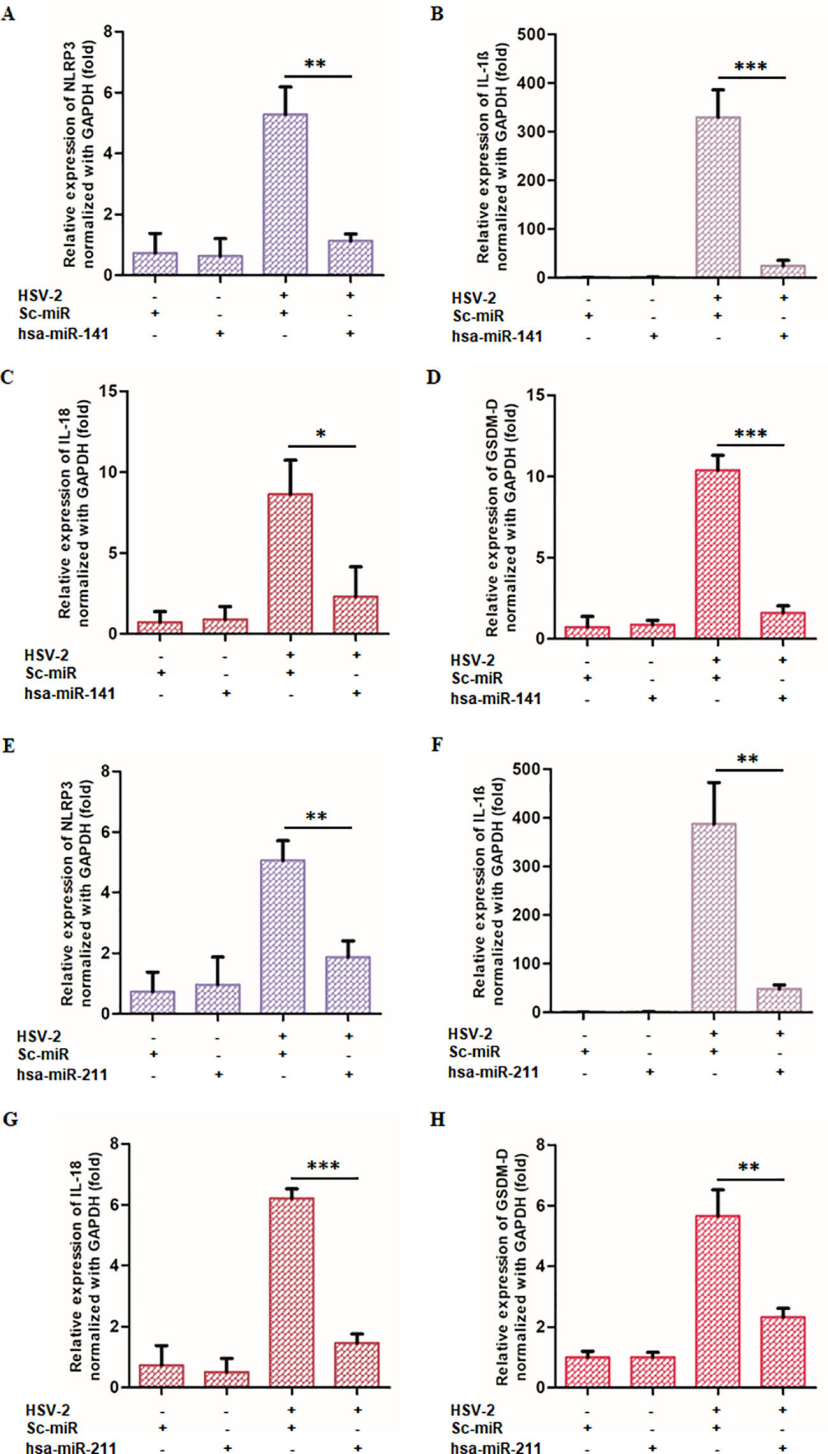
miR-141 and miR-211 suppress HSV-2-induced inflammatory responses: THP-1-derived macrophages were transfected with either miRNA or scrambled controls before HSV-2 infection. The expression of key inflammatory markers (NLRP3, GSDM-D, IL-18, and IL-1β) was quantified using qRT-PCR. **(A-D)** Effects of miR-141 on inflammatory markers. miR-141 transfection significantly reduced NLRP3, GSDM-D, IL-18, and IL-1β expression compared to HSV-2-infected controls. **(E-H)** Effects of miR-211 under identical experimental conditions. miR-211 demonstrated similar anti-inflammatory effects, reducing NLRP3, GSDM-D, IL-18, and IL-1β expression. The “-” and “+” symbols indicate absence or presence of HSV-2, sc-miR, miR-211, or miR-141 as specified. Data represent the mean ± SD of at least three independent experiments. Statistical analysis between two groups was performed using an unpaired two-tailed Student’s t-test (*p<0.05, **p<0.01, ***p<0.001).

Since NLRP3 and CASP1 are primary targets of miR-141 and miR-211, respectively, we next examined whether ectopic miRNA expression could modulate inflammasome activation during HSV-2 infection. THP-1-derived macrophages transfected with miR-141 or miR-211 mimics were infected with HSV-2 (MOI = 1), and expression levels of NLRP3, GSDM-D, IL-1β, and IL-18 were quantified by qPCR at 8 hpi (**Figure 6**).

Transfection with miR-141 mimics resulted in a significant downregulation of NLRP3 expression (78.6%), IL-1β (92.5%), IL-18 (73.2%), and GSDM-D (84.6%) compared to infected controls (**Figures 6A–D**). Similarly, miR-211 mimic transfection suppressed NLRP3 by 63.0%, IL-1β by 87.5%, IL-18 by 76.5%, and GSDM-D by 58.9% (**Figures 6E–H**).

To further confirm the proviral role of inflammasome activation in HSV-2 replication, THP-1 macrophages were pretreated with MCC950 (NLRP3 inhibitor) or Z-VAD-FMK (pan-caspase inhibitor) before infection. Both inhibitors significantly reduced HSV-2 UL30 and UL44 gene expression (**Supplementary Figure S5**) and decreased viral protein levels of glycoprotein B (gB) and ICP8, as shown by Western blot analysis (**Supplementary Figure S6**).

**Figure 7:**
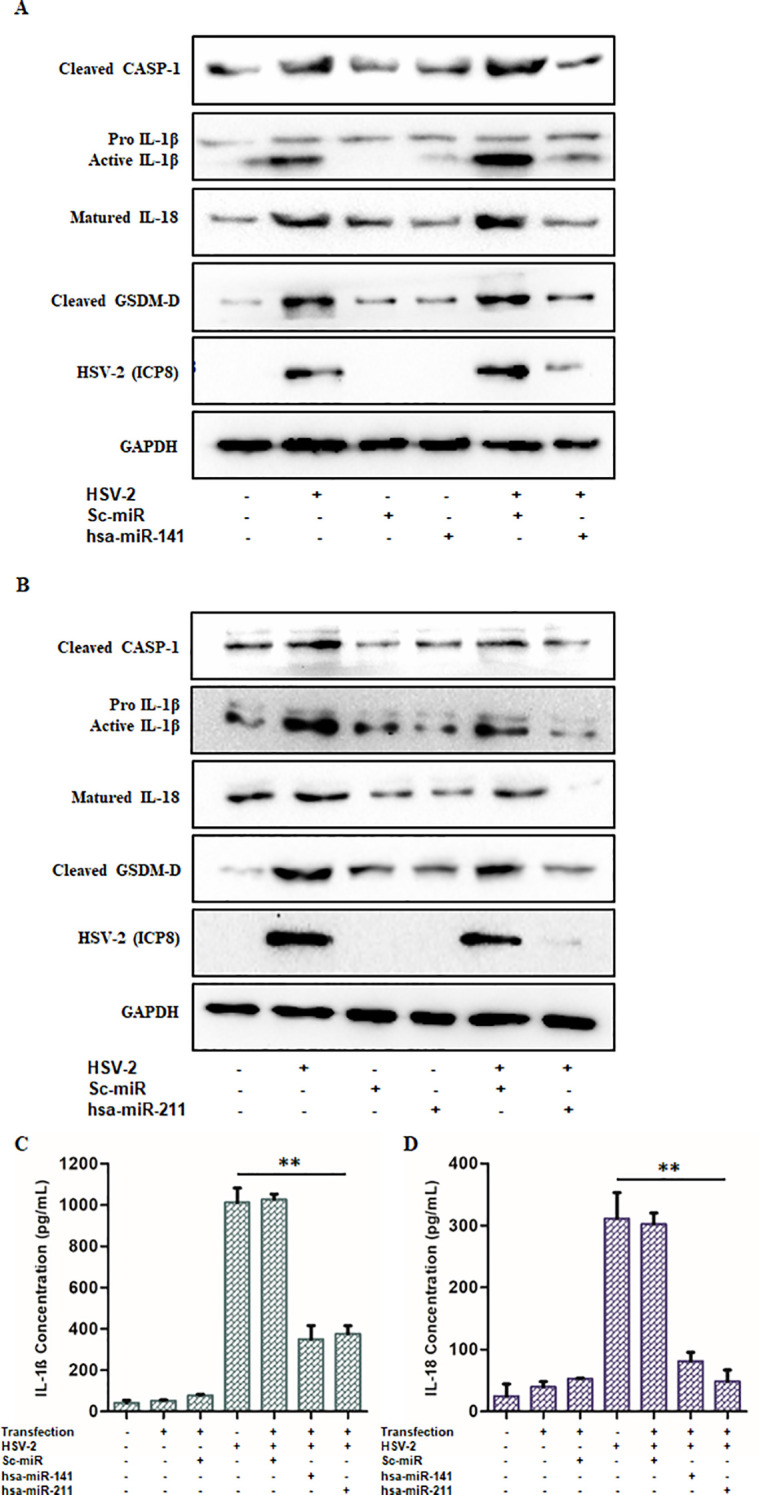
miR-141 and miR-211 exhibit anti-inflammatory effects upon HSV-2 infection: The anti-inflammatory roles of miR-141 and miR-211 were confirmed through Immunoblotting and ELISA. **(A, B)** The relative protein expression levels of the active inflammatory markers- Caspase-1, IL-1β, IL-18 and GSDM-D were observed to be significantly decreased in miR-141 and miR-211 transfected and HSV-2 infected cells as compared to untransfected but infected cells. These reduced expression levels were not observed in Sc-miR transfected cells emphasizing on the sequence-complementarity-based specific gene regulation by the respective miRNAs. HSV-2 ICP8 protein levels confirmed the progression of infection, whereas, GAPDH was used as the loading control. Furthermore, **(C, D)** The declined release of the pro-inflammatory cytokines, IL-1β and IL-18 obtained through the ELISA further evidenced the negative impact of these miRNAs on the regulation of inflammation induced by HSV-2 infection. Data represent the mean ± SD of three independent experiments. Statistical analysis was performed using one-way ANOVA followed by Tukey’s multiple comparisons test. The number of asterisks, as shown in each of the graphs, indicates the level of significance of the data (**p<0.01).

Additionally, Western blot analysis of HSV-2-infected cells transfected with miR-141 or miR-211 mimics demonstrated reduced expression of cleaved CASP1, cleaved IL-1β, IL-18, and GSDM-D proteins compared to control-transfected cells (**Figures 7A, B**). ELISA quantification of secreted IL-1β and IL-18 also revealed a significant decrease (63–66% for IL-1β and 73–84% for IL-18) in miRNA mimic-transfected cells relative to controls (**Figures 7C, D**).

Collectively, these findings demonstrate that miR-141 and miR-211 not only suppress HSV-2 replication but also mitigate inflammasome activation and pyroptotic inflammation by targeting key components of the NLRP3 inflammasome pathway. These results highlight the therapeutic potential of miRNA-based strategies in controlling HSV-2-induced immunopathology and viral dissemination.

## 4 Discussion

Herpes simplex virus type 2 is a major global health concern, contributing to significant morbidity due to its ability to establish chronic infection, cause recurrent genital ulcers, and induce severe neurological complications such as meningitis and encephalitis (Hull et al., 1984; Zhu and Viejo-Borbolla, 2021). The inflammatory pathway activation always represents a double-edged sword in viral infections (Lobo et al., 2019; Mukhopadhyay et al., 2019). The complex interplay between HSV-2 and the host immune system allows the virus to evade immune clearance while simultaneously triggering excessive inflammation that exacerbates disease pathology. The inflammatory response is primarily mediated by the activation of innate immune signaling pathways, with the NLRP3 inflammasome playing a crucial role in viral recognition and cytokine maturation (Xu and Núñez, 2023). However, while HSV-1 has been extensively studied in this context, the precise mechanisms by which HSV-2 regulates inflammasome activation remain poorly understood. This study identifies a novel miRNA-mediated regulatory mechanism in HSV-2-induced inflammation, demonstrating that HSV-2 suppresses miR-141 and miR-211 expression to enhance NLRP3 inflammasome activation, leading to increased IL-1β and IL-18 secretion, pyroptotic cell death, and viral propagation. Furthermore, restoring miR-141 and miR-211 expression significantly attenuates HSV-2-induced inflammation and viral replication, highlighting their potential as therapeutic candidates for controlling HSV-2 pathogenesis.

Our findings demonstrate that HSV-2 infection of THP-1-derived macrophages triggers a time-dependent increase in NLRP3, ASC, and caspase-1 expression, accompanied by the maturation and release of IL-1β and IL-18, hallmark cytokines of inflammasome activation. The excessive cytokine secretion observed in our study is consistent with reports on HSV-1-induced neuroinflammation, suggesting that similar mechanisms may drive HSV-2 pathogenesis (Li et al., 2019; Vitali et al., 2020). Notably, inflammasome activation often results in pyroptosis, an inflammatory form of programmed cell death that contributes to host defense but can also amplify tissue damage and viral dissemination (Guo et al., 2015; Yao et al., 2024). Our results confirm that HSV-2 infection leads to pyroptosis, as indicated by significant upregulation of Gasdermin-D (GSDM-D) cleavage and increased IL-1β secretion. These findings are in line with previous studies demonstrating that HSV-1 induces pyroptosis in microglial cells, further supporting the notion that HSV-2 exploits inflammasome activation as part of its pathogenic strategy (Hu et al., 2022; Jiang et al., 2024).

Accumulating evidence suggests that host miRNAs play a critical role in regulating inflammatory pathways, including those associated with viral infections (Piedade and Azevedo-Pereira, 2016; Banerjee et al., 2023; Dass et al., 2024). Our study identifies miR-141 and miR-211 as key negative regulators of inflammasome activation, which are significantly downregulated upon HSV-2 infection. Interestingly, miR-141 suppression was observed predominantly at early time points (8–16 hpi), whereas miR-211 was more strongly downregulated at later stages (16–24 hpi). This differential regulation suggests that HSV-2 may actively modulate host miRNA expression in a time-dependent manner to enhance inflammasome activation and facilitate viral survival. A fold change of 50% or greater downregulation in miRNA expression upon viral infection suggests that these miRNAs are involved in regulating HSV-2-induced inflammatory responses, making them potential therapeutic targets. Since miR-141 and miR-211 have been reported to negatively regulate inflammatory pathways, their suppression during HSV-2 infection may contribute to exacerbated inflammasome activation and cytokine release, thereby amplifying pathogenic inflammation. Several studies have reported that herpesviruses, including HSV-1 and Epstein-Barr virus (EBV), manipulate miRNA expression to evade immune surveillance and establish persistent infections (Wang et al., 2022; Yan et al., 2023; Long et al., 2024). Our findings suggest a similar mechanism in HSV-2 infection, wherein the suppression of miR-141 and miR-211 amplifies HSV-2-mediated inflammation, contributing to increased viral replication and immune pathology.

To establish the functional significance of miR-141 and miR-211 in inflammasome regulation, we employed *in silico* target prediction analysis and luciferase reporter assays, which confirmed that miR-141 directly targets NLRP3, while miR-211 targets caspase-1 (CASP1). These results provide strong evidence that these miRNAs function as post-transcriptional repressors of inflammasome activation. The reduction in luciferase activity observed in cells co-transfected with miRNA mimics and wild-type 3′ UTR sequences of NLRP3 and CASP1, but not with mutated constructs, confirms the specificity of these interactions. Furthermore, Western blot analysis validated that transfection with miR-141 and miR-211 mimics significantly reduced NLRP3 and caspase-1 protein levels, reinforcing their roles as negative regulators of inflammasome signaling. These findings are consistent with previous studies showing that miRNA-mediated suppression of inflammasome components can limit excessive inflammation in viral infections such as influenza and hepatitis C virus (HCV) (Boaru et al., 2015; Cao et al., 2022).

Importantly, our study also demonstrates that restoring miR-141 and miR-211 expression effectively suppresses HSV-2 replication, suggesting a dual role for these miRNAs in modulating both inflammation and viral gene expression. Transfection of THP-1-derived macrophages with miR-141 or miR-211 mimics resulted in a significant reduction in HSV-2 viral genes, including RL2, UL30, and UL44, which correspond to the immediate-early, early, and late phases of the HSV-2 replication cycle. These findings strongly suggest that miR-141 and miR-211 interfere with multiple stages of HSV-2 replication, potentially through direct or indirect regulation of viral gene expression and host immune responses. The ability of miRNAs to regulate both host immune signaling and viral propagation presents a promising avenue for therapeutic intervention, particularly in viral infections where excessive inflammation contributes to disease severity (Hum et al., 2021; Diener et al., 2022).

While our study provides compelling evidence for miR-141 and miR-211 as key regulators of HSV-2-induced inflammasome activation, several aspects warrant further investigation. The mechanisms underlying HSV-2-mediated downregulation of miR-141 and miR-211 will be explored in ongoing studies. Furthermore, the *in vivo* relevance of these findings should be validated using HSV-2 infection models to assess whether miRNA-based interventions can effectively mitigate inflammation and viral burden in physiological setting. The development of targeted miRNA delivery systems, such as lipid nanoparticles or viral vectors, could enhance the stability, bioavailability, and therapeutic efficacy of these miRNAs. Recent advances in nanoparticle-based delivery platforms have shown promising potential to improve miRNA therapeutics while minimizing off-target effects, making them an attractive strategy for future studies (Piedade and Azevedo-Pereira, 2016; Diener et al., 2022). In addition, combining miRNA-based therapies with existing antiviral agents, such as acyclovir, could be explored as a synergistic approach for the treatment of HSV-2 infections.

## 5 Conclusions

**Figure 8:**
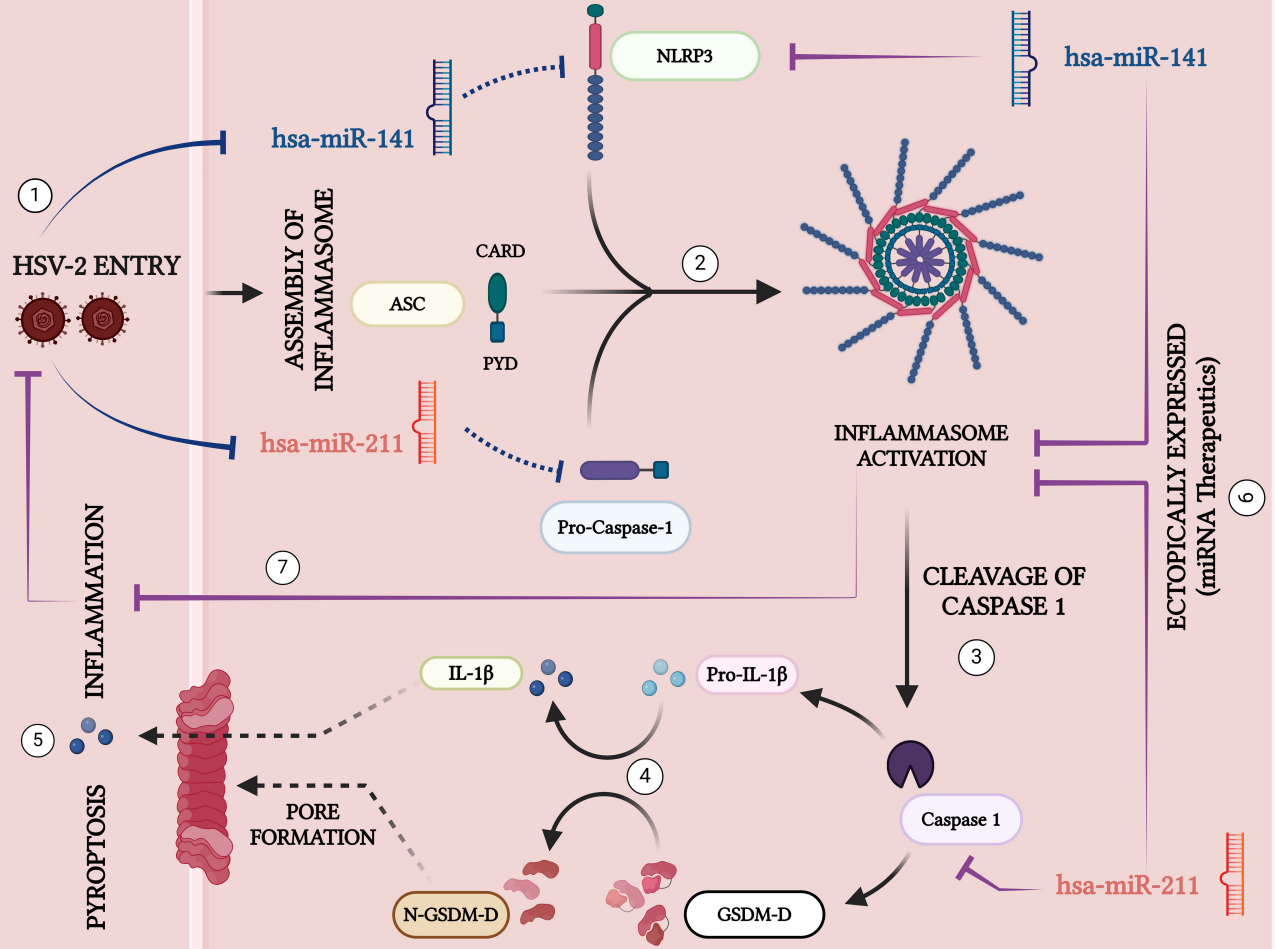
Schematic representation of the key signaling pathways involved in HSV-2-induced inflammation and pyroptosis: 1. *HSV-2 Entry:* Herpes Simplex Virus Type 2 (HSV-2) enters the host cell, initiating inflammatory responses. 2. *Inflammasome Assembly:* Upon HSV-2 infection, the miRNAs, miR-141 and miR-211 that target NLRP3 and CASP, are downregulated- a mechanism that may be crucial in facilitating the assembly of the inflammasome complex containing NLRP3, ASC, and Pro-Caspase-1. *3. Activation of Caspase-1:* The assembled inflammasome activates Caspase-1 by cleaving its precursor, pro-Caspase-1. Activated Caspase-1 facilitates downstream inflammatory responses. *4. Cytokine Maturation:* Caspase-1 in its active form cleaves the pro-inflammatory cytokine precursor Pro-IL-1β into its active form, IL-1β, which is then secreted excessively to amplify the inflammatory response. *5. Pyroptosis and Pore Formation:* Caspase-1 also converts Gasdermin-D (GSDM-D) by cleaving into its N-terminal active fragment (N-GSDM-D), forming membrane pores. This results in pyroptosis, a form of programmed cell death, and the release of excessive inflammatory cytokines. *6. Ectopic Expression of miRNAs:* MicroRNAs hsa-miR-141 and hsa-miR-211 are potential therapeutic agents. Their ectopic expressions suppress the inflammasome assembly and activation by targeting the key components NLRP3 and Caspase-1, respectively, thus mitigating inflammatory responses and viral replication. 7. *By restricting inflammasome activation*, these miRNAs limit the severity of HSV-2 infection, demonstrating their potential as therapeutic agents to inhibit inflammation and control viral pathogenesis.

Our study identifies miR-141 and miR-211 as key regulators of HSV-2-induced inflammasome activation and pyroptotic inflammation, revealing a novel miRNA-mediated mechanism in HSV-2 pathogenesis (**Figure 8**). We demonstrate that HSV-2 infection downregulates these miRNAs, leading to enhanced NLRP3 inflammasome activation, increased caspase-1 cleavage, and excessive IL-1β and IL-18 secretion, which drive pyroptotic cell death and inflammation. These findings suggest that HSV-2 actively modulates host miRNA expression to evade immune control and establish a pro-inflammatory environment favorable for viral propagation. Functional validation confirms that miR-141 directly targets NLRP3, while miR-211 regulates caspase-1, highlighting their role in restraining inflammasome activation. Restoring miR-141 and miR-211 significantly suppresses inflammasome activation, cytokine release, and HSV-2 replication, supporting their potential as therapeutic targets. Given the increasing interest in miRNA-based antiviral strategies, further studies should validate these findings in primary human macrophages and *in vivo* models while optimizing miRNA delivery systems for therapeutic applications. Combining miRNA-based therapies with existing antiviral agents could enhance treatment efficacy and mitigate HSV-2-induced inflammation. Overall, our findings provide novel insights into miRNA-mediated regulation of inflammation and viral replication, paving the way for future miRNA-based antiviral and immunomodulatory interventions that could significantly improve HSV-2 management.

## Data Availability

The original contributions presented in the study are included in the article/**Supplementary Material**. Further inquiries can be directed to the corresponding author/s.
