# Supplementary material for: MicroRNAs as regulators of NLRP3 inflammasome activation in herpes simplex virus type 2 infection
Source: Front Cell Infect Microbiol. 2025 May 29;15:1602965. doi: 10.3389/fcimb.2025.1602965 (PMC12158933; doi:10.3389/fcimb.2025.1602965)
Supplement: Supplementary file 1 [file Table1.docx]

Supplementary Material

# Supplementary Tables

# Supplementary Table S1: Primer Sequences for Inflammasome-Related Genes. This table lists the primer sequences used for amplifying genes associated with inflammasome activation and inflammatory responses.

|  | **Gene Name** | **Primer Sequences** |
| --- | --- | --- |
| **1.** | **NLRP3** | **Forward:** TCCTCGGTACTCAGCACTAAT |
|  |  | **Reverse:** AAGAGTCCCTCACAGAGTAGTT |
| **2.** | **ASC** | **Forward:** CTTATCGCGAGGGTCACAAA |
|  |  | **Reverse:** AGCTTCCGCATCTTGCTT |
| **3.** | **Caspase-1** | **Forward:** CCTGGTGTGGTGTGGTTTA |
|  |  | **Reverse:** ATCCTTCTCTATGTGGGCTTTC |
| **4.** | **IL-1β** | **Forward:** GGACAGGATATGGAGCAACAA |
|  |  | **Reverse:** CCCAAGGCCACAGGTATTT |
| **5.** | **IL-18** | **Forward:** GAAGAGGAAAGGAACCTCAGAC |
|  |  | **Reverse:** GGTTCAGCAGCCATCTTTATTC |
| **6.** | **Gasdermin D** | **Forward:** CCATCTGAGCCAGAAGAAGAC |
|  |  | **Reverse:** GACGTCCAAGTCAGAGTCAATAA |

# Supplementary Table S2: Primer Sequences for 3' UTR Regions targeted by microRNAs. This table provides details of primers designed to amplify the 3' untranslated regions (3'UTRs) of specific genes targeted by microRNAs (miRNAs). These regions are analyzed to assess miRNA binding and regulatory effects.

|  | **Gene** | **Primer Sequences** |
| --- | --- | --- |
| **1.** | **3’UTR OF NLRP3 (miR-141-3p target)** | **Forward: 5’-** CCTTCTTGGTACGCGTGGAAAC **-3’** |
|  |  | **Reverse: 5’-**GAT CGA AGC TTT CTC CAC CTG **-3’** |
| **2.** | **3’UTR OF CASPASE 1 (miR-211-5p target)** | **Forward: 5’-**AGG AAC GCG TAT GAA TGT CT**-3’** |
|  |  | **Reverse: 5’-**TGT AAA GCT TGA GTT CTT GAC TCA**- 3’** |

# Supplementary Figures


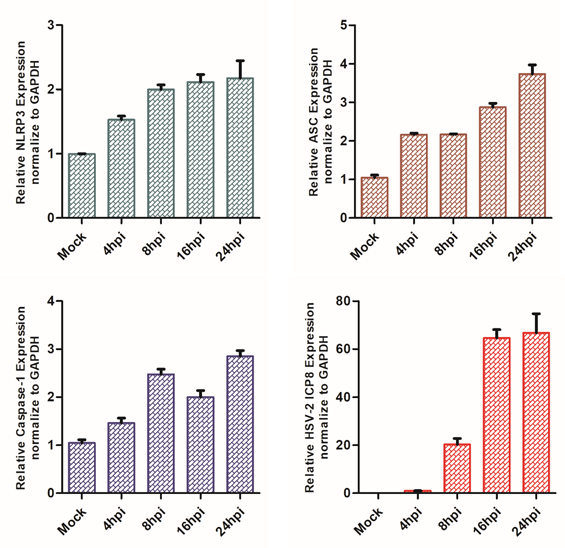


# Supplementary Figure S1 corresponding to Figure 1E: Densitometry analysis of inflammasome protein expression shown in Figure 1E. Quantification of NLRP3, ASC, and cleaved CASP1 protein levels in HSV-2-infected THP-1-derived macrophages at 4, 8, 16, and 24 hpi, normalized to GAPDH. Band intensities were measured using ImageJ software. Data represent the mean ± SEM of three independent experiments.


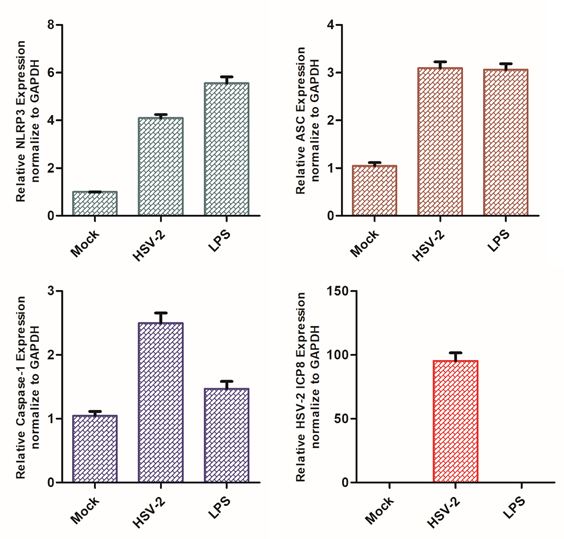


# Supplementary Figure S2 corresponding to Figure 1F: Densitometry analysis of cytokine protein expression shown in Figure 1F. Quantification of NLRP3, ASC, and cleaved CASP1 protein levels in HSV-2-infected or LPS-treated THP-1-derived macrophages normalized to GAPDH. Band intensities were determined using ImageJ software. Data represent the mean ± SEM of three independent experiments.


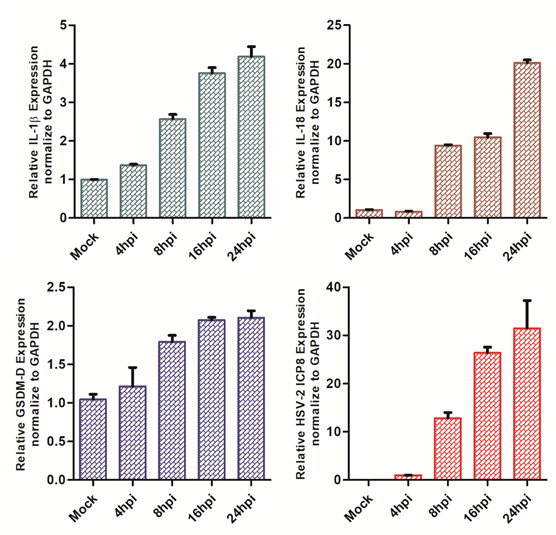


# Supplementary Figure S3 corresponding to Figure 2E: Densitometry analysis of inflammasome and pyroptosis-related protein expression shown in Figure 2E. Quantification of mature-IL-1β, -IL-18 and cleaved GSDMD protein levels in HSV-2-infected THP-1-derived macrophages at 4, 8, 16, and 24 hpi, normalized to GAPDH. Band intensities were measured using ImageJ software. Data represent the mean ± SEM of three independent experiments.


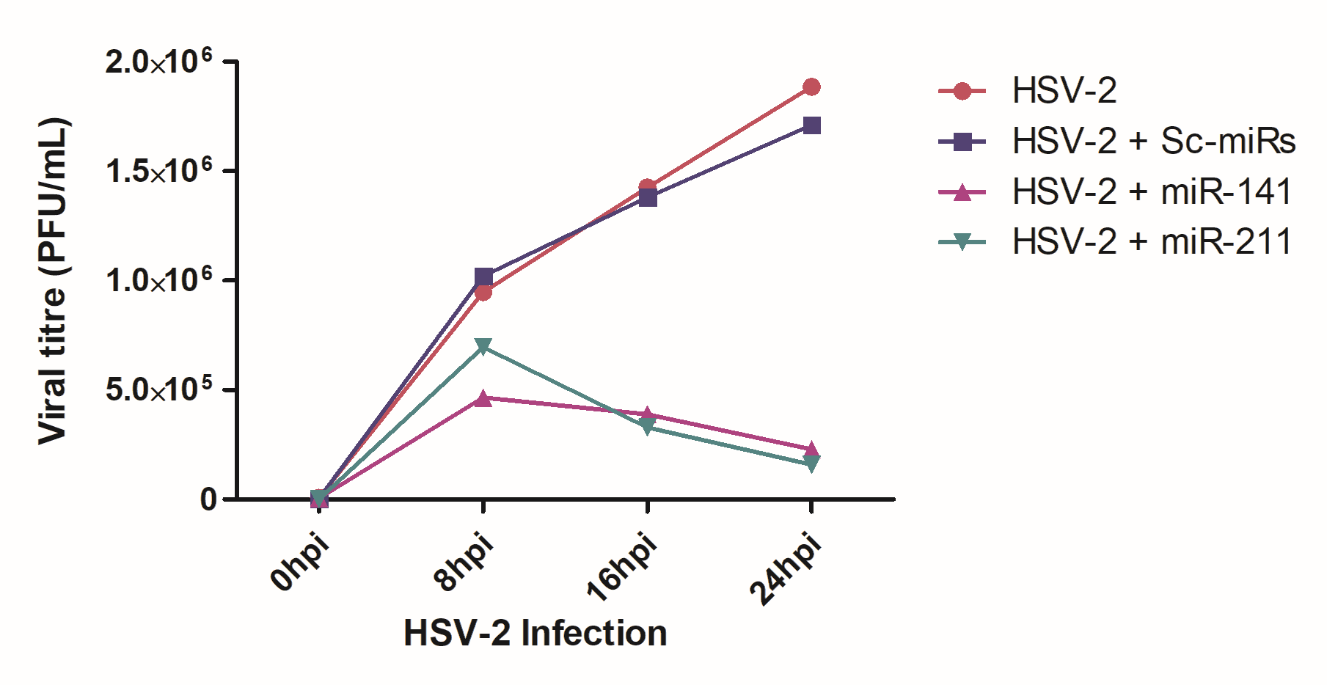


# Supplementary Figure S4 corresponding to Figure 5: THP-1-derived macrophages were transfected with miR-141 mimic, miR-211 mimic, or scrambled control mimic and subsequently infected with HSV-2. Culture supernatants were collected at indicated time points and infectious virion titers were quantified by plaque assay on Vero cells. Both miR-141 and miR-211 overexpression resulted in a significant reduction (70–85%) in infectious HSV-2 titers compared to scrambled control.


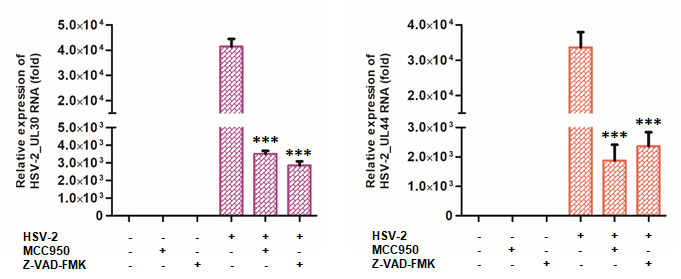


# Supplementary Figure S5 corresponding to Figure 6: THP-1-derived macrophages were pretreated with MCC950 (10 µM) or Z-VAD-FMK (50 µM) for 1 hour prior to HSV-2 infection. Relative expression levels of HSV-2 UL30 and UL44 genes were measured by qPCR at 24 hpi and normalized to GAPDH. Both MCC950 (NLRP3 inhibitor) and Z-VAD-FMK (Pan-caspase inhibitor) treatments significantly reduced HSV-2 gene expression compared to untreated infected controls. Data represent the mean ± SEM of three independent experiments. Statistical analysis was performed using one-way ANOVA followed by Tukey’s multiple comparisons test (*p < 0.05, **p < 0.01, ***p < 0.001).


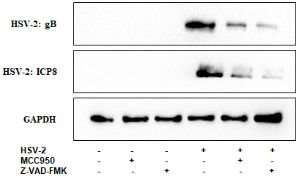


# Supplementary Figure S6 corresponding to Figure 6: THP-1-derived macrophages were pretreated with MCC950 or Z-VAD-FMK for 1 hour prior to HSV-2 infection. Western blot analysis was performed at 24 hpi to assess viral protein expression, using antibodies against HSV-2 glycoprotein B (gB) and immediate early protein ICP8. GAPDH was used as a loading control. Both inflammasome inhibitors resulted in a marked reduction in HSV-2 protein levels compared to untreated infected controls. Data are representative of three independent experiments.
